# Pregnancy outcomes according to the definition of gestational diabetes

**DOI:** 10.1371/journal.pone.0229496

**Published:** 2020-03-05

**Authors:** Sanna Koivunen, Matti Viljakainen, Tuija Männistö, Mika Gissler, Anneli Pouta, Risto Kaaja, Johan Eriksson, Hannele Laivuori, Eero Kajantie, Marja Vääräsmäki

**Affiliations:** 1 PEDEGO Research Unit, MRC Oulu, Oulu University Hospital and the University of Oulu, Finland; 2 Public Health Promotion Unit, National Institute for Health and Welfare, Helsinki and Oulu, Finland; 3 Northern Finland Laboratory Centre NordLab, Department of Clinical Chemistry and MRC Oulu, Oulu University Hospital and the University of Oulu, Oulu, Finland; 4 Information Department, National Institute for Health and Welfare, Helsinki, Finland and the Karolinska Institute, Department of Neurobiology, Care Sciences and Society, Stockholm, Sweden; 5 Department of Government Services, National Institute of Health and Welfare, Helsinki, Finland; 6 University of Tampere, Faculty of Medicine and Life Sciences, Tampere, Finland; 7 Department of General Practice, University of Helsinki and Helsinki University Hospital Folkhälsan Research Centre, Helsinki, Finland; 8 Department of Obstetrics and Gynaecology, Tampere University Hospital and University of Tampere, Faculty of Medicine and Life Sciences, Tampere, Helsinki; 9 Medical and Clinical Genetics. University of Helsinki and Helsinki University Hospital, Helsinki, Finland; 10 Institute for Molecular Medicine Finland, Helsinki Institute of Life Sciences, University of Helsinki, Helsinki, Finland; 11 Department of Clinical and Molecular Medicine, Norwegian University for Science and Technology, Trondheim, Norway; 12 Children’s Hospital, Helsinki University Hospital and the University of Helsinki, Helsinki, Finland; Stony Brook University Health Sciences Center School of Medicine, UNITED STATES

## Abstract

**Objective:**

To assess the frequency and perinatal outcomes of gestational diabetes mellitus (GDM) defined by the criteria according to the International Association of Diabetes in Pregnancy Study Group (IADPSG) and the National Institute for Health and Care Excellence (NICE) diagnostic criteria for GDM.

**Design:**

A retrospective cohort study.

**Setting:**

Six secondary and tertiary delivery hospitals in Finland in 2009.

**Population:**

Pregnant women (*N* = 4,033) and their offspring.

**Methods:**

We used data on comprehensive screening of pregnant women with a 2-h 75-g oral glucose tolerance test (OGTT), performed between gestational weeks 24 and 40. OGTT glucose concentrations were used to identify women who fulfilled IADPSG and NICE criteria. While cut-offs according to Finnish national criteria partly overlapped with both criteria, a subgroup of IADPSG- or NICE-positive GDM women remained undiagnosed by Finnish criteria and hence non-treated. They were analysed as subgroups and compared to controls who were negative with all cut-offs.

**Main outcome measures:**

GDM prevalence, birth weight SD score (BWSDS), large for gestational age (LGA) and caesarean section (CS) rates.

**Results:**

Among the 4,033 women screened for GDM, 1,249 (31.0%) and 529 (13.1%) had GDM according to the IADPSG and NICE criteria, respectively. The LGA rate was similar in both groups. Regardless of the diagnostic criteria, women with GDM had a higher risk of induced delivery and CSs than controls. In IADPSG-positive non-treated women, offspring’s BWSDS and CS rate were higher than in controls.

**Conclusions:**

GDM prevalence was 2.4-fold higher according to the IADPSG compared with the NICE criteria but the LGA rate did not differ. BWSDS and CS rate were increased already with mild untreated hyperglycaemia.

## Introduction

The prevalence of gestational diabetes mellitus (GDM) varies, depending on the screening methods and diagnostic cut-off values applied. For decades, there have been attempts to standardize the definition, but a consensus has yet to be reached. In 2010, the International Association of the Diabetes and Pregnancy Study Group (IADPSG) proposed new diagnostic criteria based on the Hyperglycemia and Adverse Pregnancy Outcomes (HAPO) study.[1,2] These guidelines recommended universal GDM screening using a 2-h 75-g oral glucose tolerance test (OGTT). The proposed cut-off values represented an odds ratio of 1.75 for birthweight > 90th centile, cord C-peptide > 90th centile (indicating neonatal hyperinsulinemia) and percent body fat > 90th centile. Importantly, for the first time, these diagnostic criteria were based on perinatal outcomes instead of the mother’s subsequent diabetes risk [3].

The diagnostic cut-off values for plasma samples according to the IADPSG criteria are ≥ 5.1 mmol/L at baseline (fasting sample), ≥ 10.0 mmol/L 1 h and ≥ 8.5 mmol/L 2 h after a glucose load. These criteria have been widely adopted and are currently recommended by the World Health Organization and the International Federation of Gynecology and Obstetrics (FIGO) [4,5]. However, the National Institutes of Health in the U.S. and the National Institute for Health and Care Excellence (NICE) in the U.K have not accepted the recommendation or diagnostic cut-offs because of concerns about a low cost-benefit ratio and limited evidence of improvements in maternal and neonatal outcomes [6–8]. Thus, at present, the NICE criteria for the diagnostic cut-offs for fasting and for 2-h postprandial glucose concentrations differ significantly from those of the IADPSG (≥ 5.6 mmol/L whichever ≥ 7.8 mmol/L, respectively), and the 1-h concentration is not included at all [2,7]. Besides these widely adopted diagnostic criteria, in some countries, including Finland, the diagnostic cut-off values for GDM were revised according to American Diabetes Association criteria in 2008 [9,10].

Given the significant differences in the cut-off values for plasma glucose levels, there are also likely differences in the frequency of GDM diagnoses and perinatal outcomes, depending on the guidelines applied. The objective of the present study was to evaluate the impact of two different diagnostic criteria for gestational diabetes mellitus, the IADPSG and the NICE guidelines, on the frequency of GDM and perinatal outcomes.

## Methods

The data were obtained from the register-based arm of the Finnish Gestational Diabetes Study, a population-based prospective cohort. [11] The study was initiated in conjunction with the introduction of new nationwide guidelines for GDM screening, diagnosis and treatment in Finland. [9,11]

### Cohort

The registry data were obtained from the Medical Birth Register (MBR), which includes data on the course and complications of pregnancy, delivery and perinatal health of the newborn, as well as International Classification of Diseases (ICD) codes for medical diagnoses of the mother and child. All pregnancies resulting in a live born infant or stillbirth at ≥ 22 gestational weeks (gw) or weighting ≥ 500 g are reported in the MBR. These data are linked to the Population Register Centre on live births and Statistics Finland on stillbirths and infant deaths. The coverage of the MBR is practically complete, and the quality of the data is high. [12, 13]

**Fig 1:**
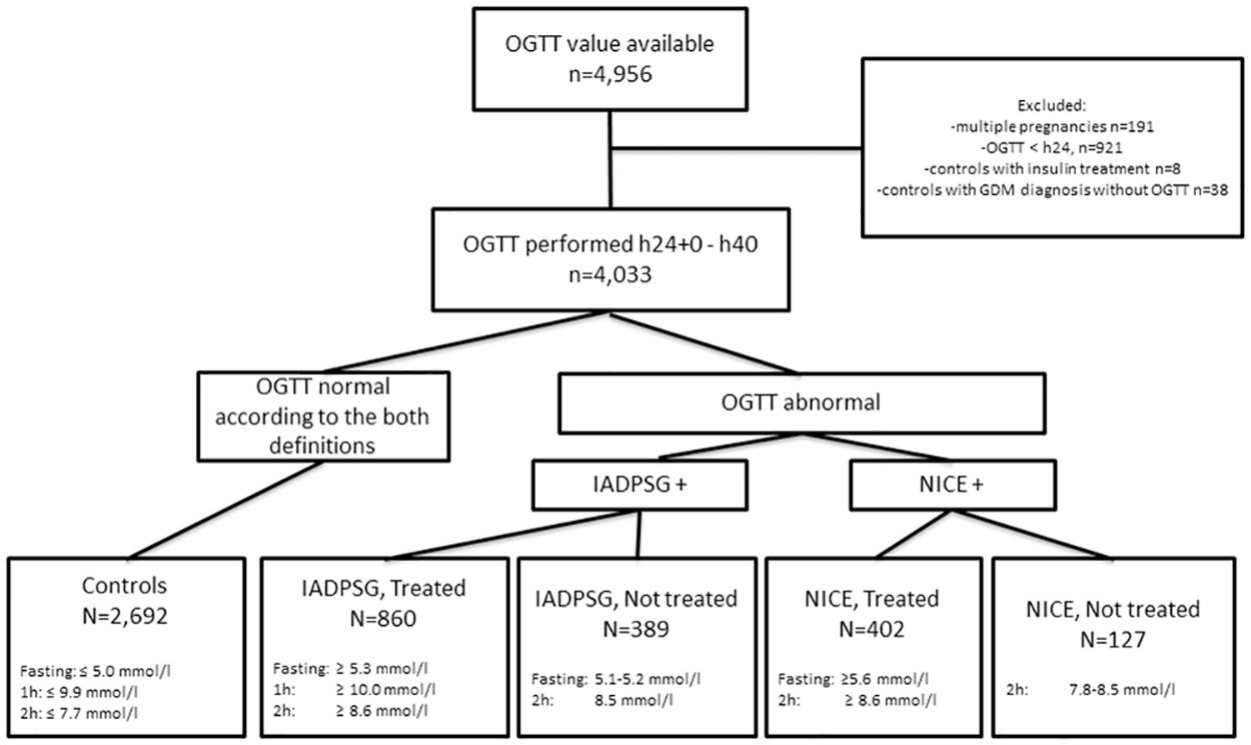
Flow chart of the study. The diagnosis of GDM was based on one abnormal value in 75g OGTT. IADPSG: International Association of the Diabetes and Pregnancy Study Group, NICE: The National Institute for Health and Care Excellence, OGTT: a 2-h 75-g oral glucose tolerance test.

The MBR also includes information on whether an OGTT was performed during pregnancy and whether the result was abnormal, but it does not include data on the actual glucose concentrations. Therefore, we collected numerical OGTT data from all women who delivered during 2009 in six delivery units in Finland: two tertiary-level (Oulu and Tampere) and four secondary-level (Lappeenranta, Seinäjoki, Kajaani and Pori) hospitals, each serving a specific geographical area. These hospitals were chosen as numerical OGTT data were available through the hospitals’ laboratory information systems. The data on OGTTs from 2008 to 2009 from the laboratory information systems were linked to clinical data from the MBR by personnel uninvolved in this study using unique personal identification numbers. After exclusion of women with pre-pregnancy diabetes and multiple pregnancies, the study population consisted of 4,033 women, to whom the OGTTs were performed between 24 and 40 gw. (Fig 1).

### GDM screening in Finland

The national guidelines published in 2008 introduced comprehensive screening for GDM and replaced the former risk-factor based screening policy in Finland. According to these guidelines, all women, except those with a very low risk, should be screened for GDM using a 75-g 2-h OGTT at 24–28 gw, with the samples obtained at baseline after an overnight fast and 1 and 2 h after the glucose load. High-risk women (i.e. women with prior GDM, a body mass index [BMI] > 35 kg/m^2^ or polycystic ovary syndrome with insulin resistance) undergo OGTT screening for the first time between 12 and 16 gw, and the test is repeated between 24 and 28 gw if the results are normal. Accordingly, women diagnosed with GDM in early pregnancy based on the OGTT were not included in the present study.

The OGTT is generally performed after a 12-h overnight fast in a laboratory near the patient’s residence. The samples are drawn from an antecubital vein into fluoride citrate tubes and analysed within 24 h in a local laboratory using commercial enzymatic assays, with the assays used varying between laboratories. The national diagnostic cut-offs were based on recommendation by the American Diabetes Association at the time of the study: ≥ 5.3 mmol/L at baseline (fasting sample), ≥ 10.0 mmol/L 1 h and ≥ 8.6 mmol/L 2 h after the glucose load (S1 Table).^9,10^ In 2009, which was the first year after the implementation of the new guidelines, 42% of all pregnant women in Finland underwent an OGTT during pregnancy. Thereafter, the coverage increased significantly, reaching 66% in 2017. According to the national guidelines, women with one or more abnormal OGTT values receive individualized dietary and lifestyle counselling and begin glucose self-monitoring. Insulin therapy at the delivery hospital is considered if self-monitored plasma glucose concentrations repeatedly exceed the target levels (< 5.5 mmol/L fasting or < 7.8 mmol/L 1 h postprandial), despite the dietary intervention. The use of oral glycemic agents was occasional and not primarily recommended by the guidelines.

### Covariates

Maternal age was defined at the time of delivery, and parity was defined as the number of previous deliveries. The BMI was calculated using self-reported height and weight before pregnancy (kg/m^2^), both of which were recorded at the first antenatal visit. Socioeconomic status was divided into four categories using the occupation reported in the MBR: upper-level employees, lower-level employees, manual workers and others, such as stay-at-home mothers, students, pensioners and self-employed individuals. Self-reported smoking status was categorized as non-smokers, those who stopped during the first trimester and those who smoked after the first trimester, as registered in the MBR.

### Outcomes

The main outcome was the frequency of GDM according to the IADPSG and NICE criteria, and the secondary outcomes were the pregnancy and neonatal outcomes in these groups. Pregnancy outcomes included pregnancy induced hypertension (ICD and Related Health Problems, version 10 [ICD 10] codes O13 and O14 included), induction of labour and delivery mode (vaginal, vacuum extraction or a caesarean section [CS]). Neonatal outcomes included birth weight, birth weight standard deviation (SD) scores, birth weight SD scores over 90%, small for gestational age (SGA) (i.e. birth weight -2 SD percentile), large for gestational age (LGA) (i.e. birth weight SD score +2 SD percentile which is the definition used in clinical practice in Finland; for comparison with other studies, we also report our results with LGA defined as birth weight SD score over 90^th^ percentile) and gestational age at delivery. The birth weight SD score is a sex-specific parameter estimating birth weight and length in singletons born at 23–43 gw to primiparous or multiparous mothers according to Finnish standards [14]. Preterm delivery was defined as a delivery prior to 37+0 gw. Because the diagnostic criteria for GDM according to current Finnish care guidelines overlap with those of the IADPSG and NICE (Table 1), a proportion of women diagnosed with mild GDM by the IADPSG (389 women, 9.6%) or NICE (127 women, 3.1%) criteria remained untreated for GDM during pregnancy. These groups were evaluated in sub-analyses (Fig 1).

**Table 1:** Diagnostic cut-off values in the 75-g OGTT according to the different diagnostic criteria.

Diagnostic method	Fasting plasma glucose mmol/L	1-h plasma glucose mmol/L	2-h plasma glucose mmol/L
IADPSG	5.1	10.0	8.5
NICE	5.6	-	7.8
Finnish guidelines*	5.3	10.0	8.6

The OGTT test was interpreted as positive for gestational diabetes if one or more values were equal to or exceeded their corresponding cut-offs.

*According to Finnish Guidelines, all pregnant women, except those with a very low risk for GDM (primiparous: age < 25 y, BMI < 25 kg/m^2^, no family history of diabetes; multiparous: age < 40 y, BMI < 25 kg/m^2^, no previous history of foetal macrosomia) are screened for GDM.

OGTT: oral glucose tolerance test

IADPSG: International Association of Diabetes in Pregnancy Study Group

NICE: National Institute for Health and Care Excellence

### Statistical analyses

All statistical analyses were carried out using the SPSS 21 statistical package. Categorical variables were reported as frequencies (%), and continuous variables were reported using the mean (SD). Pearson’s χ^2^ test was used to compare the difference in proportions in demographic variables. An independent sample *t* test was conducted to compare the difference in the means of demographic data. Differences between each GDM group were tested using Fisher’s exact test. Logistic regressions were used to estimate odds ratios (ORs), with their 95% confidence intervals (CIs) and linear regressions mean differences (with 95% CIs) of outcomes associated with GDM, respectively, according to the different diagnostic criteria and treatment status. Logistic and linear regressions were also performed to estimate differences between each GDM group. The models were adjusted for maternal age, parity and pre-pregnancy BMI. A two-sided *p* value of < 0.05 was considered statistically significant.

The study was approved by the regional ethics committee in Northern Ostrobothnia Hospital District and the National Institute for Health and Welfare. According to Finnish legislation, information consent form is not needed in Finland, when using anonymous register data only.

## Results

OGTT was performed between 24+0 and 40+0 gw (mean 27.5, SD 2.5) in 4,033 women who delivered in the study hospitals in 2009. Of these women, 1,249 (31.0%) and 529 (13.1%) had GDM according to the IADPSG and NICE criteria, respectively (Table 2). The control group consisted of 2,692 (66.7%) women who were normoglycaemic according to all criteria. Of all screened women, 860 (21.3%) had GDM according to the prevailing Finnish criteria and were counselled and medically treated for GDM, if needed. As compared with the controls, women who had GDM according to either IADPSG or NICE criteria were older and had a higher pre-pregnancy BMI. Women in the IADPSG GDM group smoked more often and were more often multiparous when compared with women the NICE GDM group. 57/860 women received insulin treatment. This represents 6.6% of women who were diagnosed by Finnish criteria. Of those who met IADPSG criteria, 4.6% received insulin, and of those who met NICE criteria, 7.2% (Table 3).

**Table 2:** Characteristics of pregnancies with and without GDM, classified according to the different criteria based on the OGTT at 24 to 40 gw.

Characteristics	No GDM according to all criteria	GDM by IADPSG criteria		GDM by NICE criteria	
			*p-*value		*p*-value
*N* (%)	2,692 (66.7)	1,249 (31.0)		529 (13.1)	
Maternal age, y	29.4 (5.3)	30.2 (5.6)	<0.001	30.3 (5.8)	<0.001
Pre-pregnancy BMI, kg/m^2^	25.5 (4.3)	27.2 (5.1)	<0.001	27.0 (5.1)	<0.001
Primiparity	1,333 (49.5)	560 (44.8)	0.006	259 (49.0)	0.815
**Smoking**					
No	2,265 (87.5)	1,017 (84.5)	0.011	435 (85.0)	0.120
Quit in the first trimester	109 (4.2)	59 (4.9)	0.336	26 (5.1)	0.379
Continued after the first trimester	215 (8.3)	128 (10.6)	0.020	51 (10.0)	0.221
**Socioeconomic status**					
Upper-white collar worker^a^	470 (21.6)	190 (19.2)	0.112	74 (17.6)	0.063
Lower-white collar worker^b^	889 (40.9)	421 (42.5)	0.417	181 (43.1)	0.413
Blue-collar worker^c^	358 (16.5)	184 (18.6)	0.151	83 (19.8)	0.102
Other^d^	454 (20.9)	196 (19.8)	0.464	82 (19.5)	0.520

Data are n (%) or mean (SD).

^a^Administrative, managerial, professional and related occupations.

^b^Administrative and clerical occupations.

^c^Manual labourer.

^d^Students, pensioners, self-employed and others.

BMI: body mass index

**Table 3:** Outcomes of pregnancies with GDM classified according to the different criteria based on the OGTT at 24 to 40 gw.

Characteristics	No GDM according to all criteria	IADPSG		NICE	
		*N* (%/SD)	*p*-value*	*N* (%/SD)	*p*-value*
*N* (%)	2,692 (66.7)	1,249 (31.0)		529 (13.1)	
Gestational age at delivery, wk	39.9 (1.6)	39.6 (1.8)	<0.001	39.4 (2.0)	<0.001
Birth weight, g (SD)	3,571 (523.7)	3,558 (557.5)	0.511	3490 (620.0)	0.002
Birth weight, SD score	-0.05 (1.0)	0.04 (1.0)	0.012	0.01 (1.1)	0.293
Small for gestational age, <-2 SD	76 (2.8)	32 (2.6)	0.640	15 (2.8)	0.987
Large for gestational age, >+2SD	72 (2.7)	36 (2.9)	0.710	21 (4.0)	0.104
Large for gestational age, >90%	250 (9.3)	141 (11.3)	0.050	65 (12.3)	0.034
Induced delivery	414 (15.4)	259 (20.7)	<0.001	121 (22.9)	<0.001
Pre-term birth	22 (0.8)	18 (1.4)	0.069	11 (2.1)	0.008
Insulin treatment	0	57 (4.6)	<0.001	38 (7.2)	<0.001
Pregnancy induced hypertension*	165 (6.1)	100 (8.0)	0.029	48 (9.1)	0.013
**Type of delivery**					
Vaginal non-instrumental	2,048 (76.1)	894 (71.6)	0.003	372 (70.3)	0.005
Instrumental	242 (9.0)	95 (7.6)	0.148	40 (7.6)	0.288
Caesarean section	402 (14.9)	260 (20.8)	<0.001	117 (22.1)	<0.001
Hospital stay of mother in days	3.1 (1.4)	3.2 (1.4)	0.001	3.4 (1.5)	<0.001
Hospital stay of offspring in days	3.1 (2.7)	3.3 (1.9)	0.025	3.4 (2.5)	0.017

Data are *n* (%) or mean (SD)

*p-value between GDM-group and controls.

**International classification of diseases ICD-10: O13, O14

When the pregnancy outcomes of the GDM groups were compared with those of the controls, the rates of labour induction, pregnancy induced hypertension was more common and CSs were higher in both GDM groups, and the gestational age at delivery was lower. In the NICE group, the proportion of pre-term deliveries was higher than that in the IADPSG group and in the controls (Table 3). The LGA rate in the two GDM groups did not differ from that in the controls.

**Fig 2:**
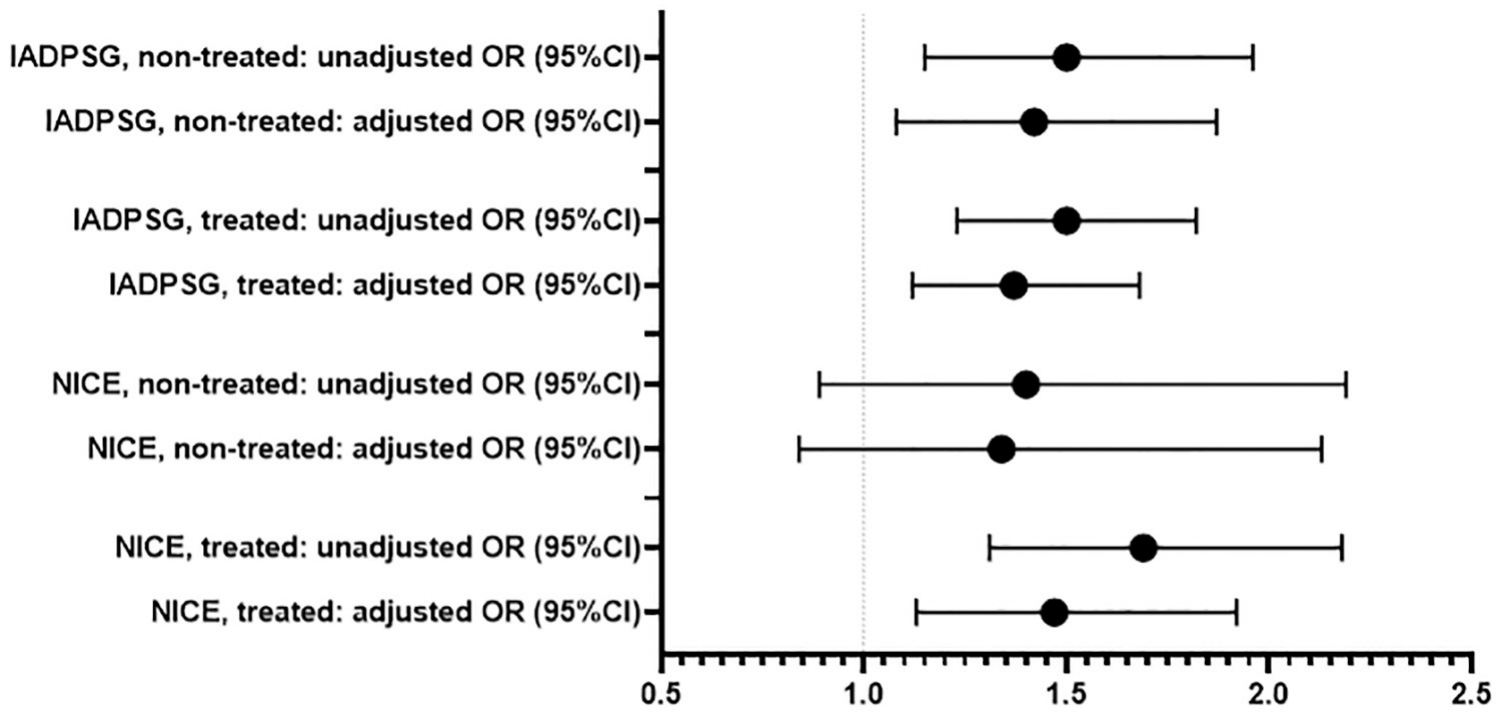
Association between caesarean section and GDM groups identified by different diagnostic criteria. Logistic regression analyses were used to estimate unadjusted and adjusted (for maternal age, parity and pre-pregnancy body mass index) odds ratios (OR) with 95% CI, whiskers expressing the 5th and 95th percentiles. IADPSG: International Association of the Diabetes and Pregnancy Study Group, NICE: The National Institute for Health and Care Excellence.

**Fig 3:**
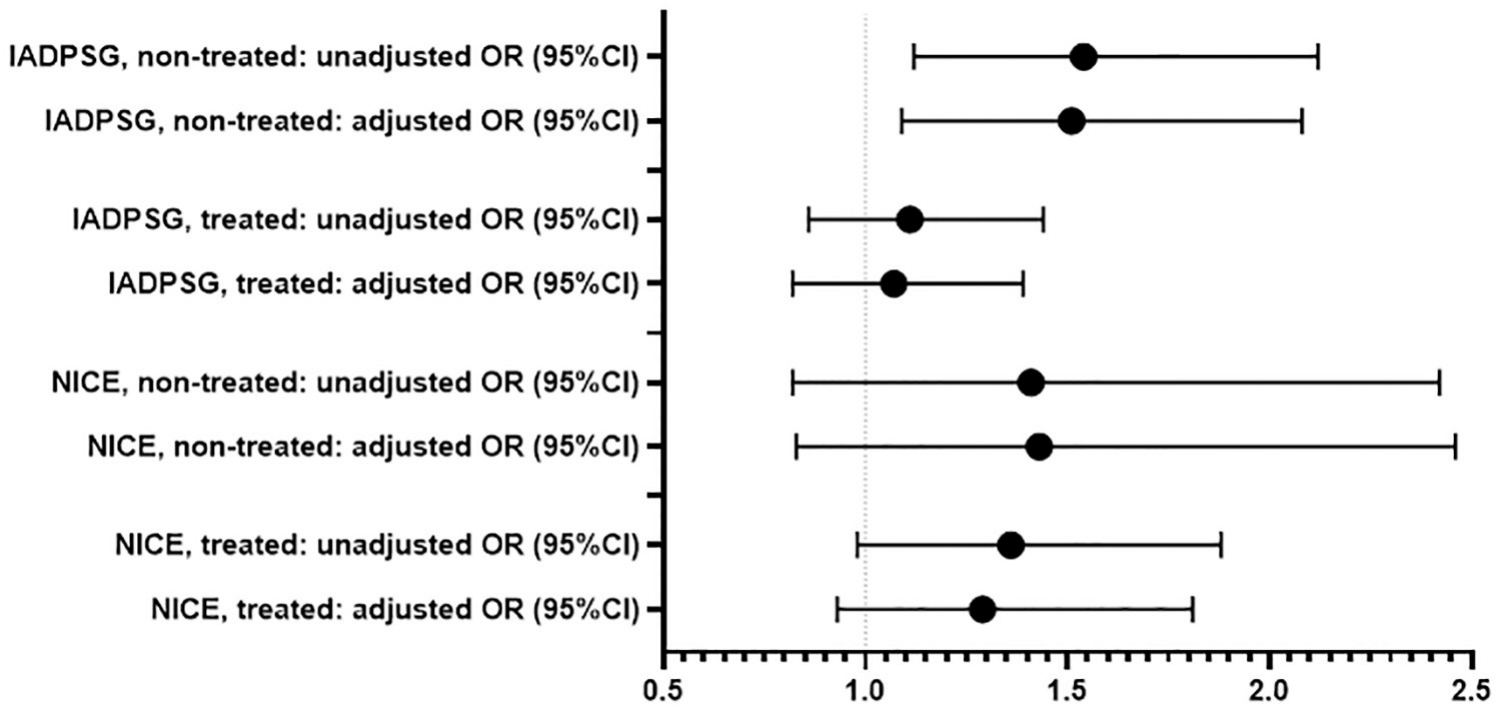
Association between large for gestational age, defined as birth weight >90th percentile, in GDM groups identified by different diagnostic criteria. Logistic regression analyses were used to estimate unadjusted and adjusted (for maternal age, parity and pre-pregnancy body mass index) odds ratios (OR) with 95% CI, whiskers expressing the 5th and 95th percentiles. IADPSG: International Association of the Diabetes and Pregnancy Study Group, NICE: The National Institute for Health and Care Excellence.

In the IADPSG and NICE groups, 68.9 and 76.0% of women, respectively, fulfilled national Finnish diagnostic criteria and thus received counselling and treatment. The characteristics of these pregnancies are presented in the Table 4. Proportion of insulin treated women was higher in the treated NICE group than in the treated IADPSG group, 9.5% and 6.6%, respectively. The delivery induction and CS rates in both treated GDM groups were higher than those in the controls. The CS rate and birth weight SD score and large for gestational age as defined at >90%, were higher in the non-treated IADPSG group than in controls (Table 5, Figs 2 and 3).

**Table 4:** Characteristics of pregnancies with GDM classified according to the different GDM diagnostic criteria, with or without treatment.

Characteristics	Control group	IADPSG				*p*-value**	NICE				*p*-value**
		Treated		Non-treated			Treated		Non-treated		
			*p*-value*		*p*-value*			*p*-value*		*p*-value*	
*N*	2,692 (66.7)	860 (21.3)		389 (9.6)			402 (10.0)		127 (3.1)		
Maternal age, y	29.4 (5.3)	30.2 (5.6)	<0.001	30.0 (5.7)	0.033	0.462	30.4 (5.9)	0.001	30.0 (5.5)	0.223	0.498
Pre-pregnancy BMI, kg/m^2^	25.5 (4.3)	27.4 (5.2)	<0.001	26.9 (4.7)	<0.001	0.150	27.4 (5.1)	<0.001	25.4 (4.6)	0.799	<0.001
Primiparity	1,333 (49.5)	378 (44.0)	0.004	182 (46.8)	0.314	0.351	193 (48.0)	0.573	66 (52.0)	0.589	0.437
**Smoking**											
No	2,265 (87.5)	691 (83.9)	0.008	326 (85.8)	0.354	0.390	326 (83.8)	0.044	109 (88.6)	0.710	0.193
Quit in the first trimester	109 (4.2)	39 (4.7)	0.521	20 (5.3)	0.347	0.692	19 (4.9)	0.541	7 (5.7)	0.428	0.722
Continued after the first trimester	215 (8.3)	94 (11.4)	0.007	34 (8.9)	0.673	0.198	44 (11.3)	0.050	7 (5.7)	0.302	0.070
**Socioeconomic status**											
Upper-white collar worker^a^	470 (21.6)	125 (18.4)	0.067	65 (20.9)	0.764	0.350	62 (19.5)	0.382	12 (11.8)	0.017	0.074
Lower-white collar worker^b^	889 (40.9)	294 (43.2)	0.291	127 (40.8)	0.970	0.478	141 (44.3)	0.252	40 (39.2)	0.728	0.363
Blue-collar worker^c^	358 (16.5)	136 (20.0)	0.035	48 (15.4)	0.638	0.086	56 (17.6)	0.616	27 (26.5)	0.009	0.051
Other^d^	454 (20.9)	125 (18.4)	0.152	71 (22.8)	0.439	0.103	59 (18.6)	0.332	23 (22.5)	0.692	0.376

Data are *n* (%) or mean (SD).

*p-value between GDM-group and controls.

**p-value between treated/non-treated.

^a^Administrative, managerial, professional and related occupations.

^b^ Administrative and clerical occupations.

^c^Manual labourer.

^d^Students, pensioners, self-employed and others.

BMI: body mass index

**Table 5:** Outcomes of pregnancies with GDM classified according to the different GDM diagnostic criteria, with or without treatment.

Characteristics	No GDM	IADPSG				*p*-value**	NICE				*p*-value**
		Treated		Non-treated			Treated		Non-treated		
			*p*-value*		*p*-value*			*p*-value*		*p*-value*	
*N*	2,692 (66.7)	860 (21.3)		389 (9.6)			402 (10.0)		127 (3.1)		
Gestational age at delivery, wk	39.9 (1.6)	39.5 (1.9)	<0.001	39.9 (1.6)	0.377	0.001	39.3 (2.0)	<0.001	39.5 (2.0)	0.002	0.339
Birth weight, g	3,571 (524)	3,530 (558)	0.061	3,620 (552)	0.095	0.008	3,499 (624)	0.013	3,463 (610)	0.025	0.565
Birth weight, SD score	-0.046 (1.0)	0.017 (1.0)	0.120	0.099 (1.1)	0.012	0.208	0.037 (1.2)	0.176	-0.074 (1.1)	0.768	0.317
Small for gestational age, <-2 SD	76 (2.8)	20 (2.3)	0.433	12 (3.1)	0.772	0.432	10 (2.5)	0.703	5 (3.9)	0.463	0.391
Large for gestational age, >+2 SD	72 (2.7)	23 (2.7)	1.000	13 (3.3)	0.453	0.514	17 (4.2)	0.082	4 (3.1)	0.747	0.587
Large for gestational age, >90%	250 (9.3)	88 (10.2)	0.411	53 (13.6)	0.007	0.079	49 (12.2)	0.066	16 (12.6)	0.212	0.903
Induced delivery	414 (15.4)	192 (22.3)	<0.001	67 (17.2)	0.349	0.039	94 (23.4)	<0.001	27 (21.3)	0.075	0.619
Preterm birth	22 (0.8)	13 (1.5)	0.073	5 (1.3)	0.354	0.756	7 (1.7)	0.073	4 (3.1)	0.007	0.332
Insulin treatment	0	57 (6.6)	<0.001	0		<0.001	38 (9.5)	<0.001	0		<0.001
Pregnancy induced hypertension***	165 (6.1)	75 (8.7)	0.008	25 (6.4)	0.820	0.167	38 (9.5)	0.012	10 (7.9)	0.426	0.589
**Type of delivery**											
Vaginal	2,048 (76.1)	616 (71.6)	0.009	278 (71.5)	0.048	0.953	278 (69.2)	0.003	94 (74.0)	0.595	0.296
Instrumental	242 (9.0)	65 (7.6)	0.193	30 (7.7)	0.406	0.924	32 (8.0)	0.498	8 (6.3)	0.297	0.537
Caesarean section	402 (14.9)	179 (20.8)	<0.001	81 (20.8)	0.003	0.997	92 (22.9)	<0.001	25 (19.7)	0.144	0.447
Hospital stay of mother in days	3.1 (1.4)	3.2 (1.4)	0.001	3.2 (1.3)	0.196	0.237	3.4 (1.5)	<0.001	3.3 (1.3)	0.033	0.615
Hospital stay of offspring in days	3.1 (2.7)	3.4 (2.1)	0.021	3.2 (1.3)	0.253	0.157	3.5 (2.8)	0.008	3.2 (1.3)	0.718	0.058

Data are n (%) or mean (SD).

*p-value between GDM-group and controls.

**p-value between treated/non-treated.

***International classification of diseases ICD-10: O13, O14

The associations we found were also present after adjustment for maternal age, parity and pre-pregnancy BMI, although most were slightly attenuated (Table 6).

**Table 6:** Multivariate logistic and linear regression analysis of perinatal and neonatal outcomes in patients according to the application of the different GDM diagnostic methods.

	Non-treated IADPSG		Treated IADPSG		Non-treated NICE		Treated NICE	
Outcome	OR	aOR	OR	aOR	OR	aOR	OR	aOR
Induction of labour	1.15 (0.86–1.52)	1.10 (0.83–1.47)	1.58 (1.31–1.92)	1.46 (1.20–1.78)	1.49 (0.96–2.30)	1.48 (0.95–2.30)	1.68 (1.30–2.16)	1.53 (1.18–1.99)
Caesarean section	1.50 (1.15–1.96)	1.42 (1.08–1.87)	1.50 (1.23–1.82)	1.37 (1.12–1.68)	1.40 (0.89–2.19)	1.34 (0.84–2.13)	1.69 (1.31–2.18)	1.47 (1.13–1.92)
Birth weight SD score	0.14 (0.04–0.25)	0.14 (0.03–0.25)	0.06 (-0.02–0.14)	0.05 (-0.03–0.13)	-0.03 (-0.21–0.15)	-0.02 (-0.20–0.16)	0.08 (-0.03–0.19)	0.07 (-0.04–0.18)
Small for gestational age, <2SD	1.10 (0.59–2.03)	1.18 (0.63–2.20)	0.82 (0.50–1.35)	0.82 (0.49–1.37)	1.41 (0.56–3.55)	1.45 (0.58–3.68)	0.88 (0.45–1.71)	0.83 (0.41–1.68)
Large for gestational age, >2SD	1.26 (0.69–2.29)	1.17 (0.64–2.14)	1.00 (0.62–1.61)	0.87 (0.53–1.43)	1.18 (0.43–3.29)	1.18 (0.42–3.30)	1.61 (0.94–2.76)	1.43 (0.82–2.50)
Large for gestational age, >90%	1.54 (1.12–2.12)	1.51 (1.09–2.08)	1.11 (0.86–1.44)	1.07 (0.82–1.39)	1.41 (0.82–2.42)	1.43 (0.83–2.46)	1.36 (0.98–1.88)	1.29 (0.93–1.81)

Data are OR (95% CI), aOR = OR adjusted for maternal age, parity and pre-pregnancy BMI. Exception: Mean difference (95% CI = confidential interval) for birth weight SD score.

## Discussion

### Main findings

Among all the women who underwent OGTT, the proportion of GDM was 2.4-fold higher when diagnosed by the IADPSG (31.0%) criteria as compared with when diagnosed by the NICE criteria (13.1%). The proportion of LGA infants was similar in both the GDM groups and controls, which may reflect successful counselling and treatment. While the diagnostic cut-offs partly overlapped, we had also a possibility to evaluate a subgroup without treatment. Mild untreated hyperglycaemia was associated with an increased CS rate and higher birth weights, as found in the HAPO study and some recent studies [15,16].

The main short-term goal of GDM management is to prevent macrosomia. Large randomized studies have demonstrated that achievement of euglycaemia leads to better perinatal outcomes such as lower birth weight and lower macrosomia rate already in mild cases of GDM. [17, 18] In our, and also some previous studies, GDM treatment can be considered successful, as neither birth weight nor perinatal outcomes differed between the controls and GDM groups [19]. The success of GDM treatment may also be attributed to the widespread adoption of national guidelines in Finland and the existence of good co-operation between primary and special health care services. The rates of induced deliveries and CSs were higher in both the treated GDM groups, irrespective of which diagnostic criteria were applied. It may be supposed that the diagnosis of GDM itself at some extent predispose women to those interventions. On the other hand, the birth weight SD score and CS rate showed an increasing tendency in cases with mild hyperglycaemia, without a diagnosis of GDM. This finding is in accordance with the results of the HAPO study, which reported a linear increase in adverse perinatal outcomes without specific cut-offs. [1]

Different diagnostic cut-offs in GDM screening might be expected to result in different maternal profiles: The IADPSG criteria emphasize the importance of fasting glucose, whereas the NICE guidelines focus on low 2-h postprandial glucose concentration. As compared with the control group, women in the IADPSG group with lower fasting glucose concentrations were more often multiparous and smokers. In the NICE GDM group, the proportion of insulin-treated mothers (7.2%) was higher than that in the IADPSG GDM group (4.6%), which may partly indicate the different grades of severity of GDM in the two groups. Between the treated groups, the difference between NICE and IADPSG groups was even higher, 9.5% vs 6.6%. Pre-term birth was most common in the NICE groups, regardless of treatment. The reason remained unclear.

In multivariate logistic and linear regression analyses the results were adjusted with maternal age, parity and pre-pregnancy BMI, which are independent risk factors for adverse pregnancy outcome. In our study, the significance of the results remained despite adjustment with these factors.

### Strengths and limitations

The strengths of this study are the large cohort and ability to evaluate the significance of numerical OGTT values. The quality and completeness of the MBR are high, and the current data cover geographically diverse regions of Finland. As a limitation, during the study period the coverage of new nationwide GDM screening was rather low (42%) while the new screening protocol had been launched just a year earlier and therefore not fully implemented. Control population consisted of those with known OGTT results. Thus, non-screened women with the lowest risk of GDM were not included in this study, but this is not a bias because we classified women with known results of OGTT, not with the risk of GDM. We speculate that those who did not undergo OGTT and the undiagnosed GDM mothers would on average represent a milder end of the GDM spectrum and the present study may underestimate their proportion. In order to exclude possible overt or pre-pregnancy diabetes, only women with OGTT performed after recommended screening time point (> 24 gestational weeks) were included. The study also had limited power to assess rare perinatal outcomes.

## Conclusion

In conclusion, there was a significant difference in the prevalence of GDM diagnosed by the different criteria, but the pregnancy outcomes were similar using the two diagnostic methods: the use of NICE criteria only would have identified less GDM women than the use of IADPSG criteria, but would also likely to have left unidentified a group of GDM women who had a similar proportion of these pregnancy outcomes and might have benefited from GDM treatment. The main aim of GDM treatment, prevention of macrosomia, was attained by both criteria. The similarity in pregnancy outcomes in GDM mothers and normoglycaemic controls may reflect successful, uniform counselling and treatment and well-organized maternal health care. Mild untreated hyperglycaemia was associated with an increased CS rate and higher birth weights, as reported by the HAPO and some recent studies. [1,20] In the future, studies are needed to determine how these different diagnostic methods will predict a woman´s subsequent risk for type 2 diabetes and other long-term outcomes.

## Supporting information

S1 Table Diagnostic threshold values in the 75-g OGTT according to the different screening criteria. (DOCX)
