# Supplementary material for: Pregnancy outcomes according to the definition of gestational diabetes
Source: PLoS One. 2020 Mar 5;15(3):e0229496. doi: 10.1371/journal.pone.0229496 (PMC7058278; doi:10.1371/journal.pone.0229496)
Supplement: S1 Table — (DOCX) [file pone.0229496.s001.docx]

**Table S1.** Diagnostic threshold values in the 75-g OGTT according to the different screening criteria.

| **Screening method** | **Fasting plasma glucose mmol/L** | **1-h plasma glucose mmol/L** | **2-h plasma glucose mmol/L** |
| --- | --- | --- | --- |
| IADPSG | 5.1 | 10.0 | 8.5 |
| NICE | 5.6 | - | 7.8 |
| Finnish guidelines* | 5.3 | 10.0 | 8.6 |

The OGTT test was interpreted as positive for gestational diabetes if one or more values were equal to or exceeded their corresponding thresholds.

*According to Finnish Guidelines, all pregnant women, except those with a very low risk for GDM (primiparous: age < 25 y, BMI < 25 kg/m^2^, no family history of diabetes; multiparous: age < 40 y, BMI < 25 kg/m^2^, no previous history of foetal macrosomia) are screened for GDM.

OGTT: oral glucose tolerance test

IADPSG: International Association of Diabetes in Pregnancy Study Group

NICE: National Institute for Health and Care Excellence
